# Epigenetic Mechanism of 5-HT/NE/DA Triple Reuptake Inhibitor on Adult Depression Susceptibility in Early Stress Mice

**DOI:** 10.3389/fphar.2022.848251

**Published:** 2022-03-17

**Authors:** Ping Meng, Chunmei Li, Sijin Duan, Shengmin Ji, Yangyang Xu, Yutong Mao, Hongbo Wang, Jingwei Tian

**Affiliations:** School of Pharmacy, Key Laboratory of Molecular Pharmacology and Drug Evaluation (Yantai University), Ministry of Education, Collaborative Innovation Center of Advanced Drug Delivery System and Biotech Drugs in Universities of Shandong, Yantai University, Yantai, China

**Keywords:** DNA methylation, triple reuptake inhibitors, second stress, depression susceptibility, epigenetics

## Abstract

Major depressive disorder (MDD) is a chronic, remitting and debilitating disease and the etiology of MDD is highly complicated that involves genetic and environmental interactions. Despite many pharmacotherapeutic options, many patients remain poorly treated and the development of effective treatments remains a high priority in the field. LPM570065 is a potent 5-hydroxytryptamine (5-HT), norepinephrine (NE) and dopamine (DA) triple reuptake inhibitor and both preclinical and clinical results demonstrate significant efficacy against MDD. This study extends previous findings to examine the effects and underlying mechanisms of LPM570065 on stress vulnerability using a “two-hit” stress mouse model. The “two-hit” stress model used adult mice that had experienced early life maternal separation (MS) stress for social defeat stress (SDS) and then they were evaluated in three behavioral assays: sucrose preference test, tail suspension test and forced swimming test. For the mechanistic studies, methylation-specific differentially expressed genes in mouse hippocampal tissue and ventral tegmental area (VTA) were analyzed by whole-genome transcriptome analysis along with next-generation bisulfite sequencing analysis, followed by RT-PCR and pyrophosphate sequencing to confirm gene expression and methylation. LPM570065 significantly reversed depressive-like behaviors in the mice in the sucrose preference test, the tail suspension test, and the forced swimming test. Morphologically, LPM570065 increased the density of dendritic spines in hippocampal CA1 neurons. Hypermethylation and downregulation of oxytocin receptor (*Oxtr*) in the hippocampal tissues along with increased protein expression of Dnmt1 and Dnmt3a in mice that experienced the “two-hit” stress compared to those that only experienced adulthood social defeat stress, and LPM570065 could reverse these changes. Combined, these results suggest that methylation specificity of the gene *Oxtr* in the hippocampus may play an important role in early life stress-induced susceptibility to depression and that the5-HT/NE/DA triple reuptake inhibitor LPM570065 may reduce depression susceptibility via the reversal of the methylation of the gene *Oxtr*.

## Introduction

Major depressive disorder is an important cause of human suffering, illness, and disability worldwide (Bromet et al., 2011; Gaebel et al., 2017). The World Health Organization ranks depression as the third leading cause of the global burden of disease and predicts that the disorder will rank first by 2030, with surveys showing that almost one in five people will experience a depressive episode at some point in their lives. The complex etiology of depression has not yet been fully elucidated and may involve genetic, environmental factors and their interactions. Abundant evidence suggests that exposure to early life stress (ELS) increases the risk of depression and may also lead to persistent changes in neural structure and depression-like behavior in adulthood. (Caspi et al., 2003; de Lima et al., 2011; Nemeroff and Binder, 2014). The experience of adversities during this critical period has lifelong impacts on the brain and behavior (Doherty et al., 2017).

Increasing evidence shows that early life stress increases susceptibility to acquired social failure and a “two-hit” model can mimic this adversity where mice first experienced early-life stress such as maternal separation (“first hit”) and then mice were exposed to repeated social defeat stress (“second hit”) (Peña et al., 2017). This manipulation causes long-lasting transcriptional and epigenetic alterations that prime the ventral tegmental area (VTA) to be in a depression-like state (Barnett Burns et al., 2018; Reshetnikov et al., 2021). So far, many epigenetic modification mechanisms have been discovered and DNA methylation is one of the most well-studied mechanisms that is believed to be involved in depression (Uddin et al., 2013; Bakusic et al., 2016; Chen et al., 2017). Early life stress can epigenetically modify depression-related genes by affecting DNA methylation, which in turn could cause structural and functional changes in the brain (Ding and Dai, 2019). Additionally, some antidepressants can epigenetically alter certain signaling molecules beyond their traditionally-believed pharmacological mechanisms to contribute to their antidepressant efficacies. For example, the antidepressant fluoxetine can epigenetically alter the CaMKIIα promotor in nucleus accumbens to regulate ΔFosB binding, which represents a new epigenetic mechanism of antidepressant action independent of its serotonin reuptake inhibition (Robison et al., 2014).

**FIGURE 1:**
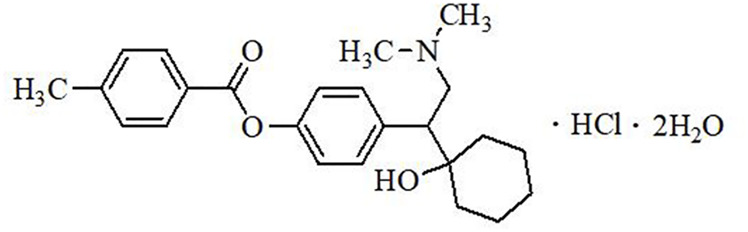
Chemical structure of LPM570065.

LPM570065 (also known as LY03005, ansofaxine, and toludesvenlafaxine) (Figure 1) is a new chemical entity and a 5-HT/NE/DA triple reuptake inhibitor. LPM570065 exhibits high binding affinity to serotonin transporter (SERT), norepinephrine transporter (NET) and dopamine transporter (DAT), and increases the release of 5-HT, NE and DA in the striatum after oral administration (Zhu et al., 2021). In several preclinical models including the forced swimming test, the chronic unpredictable mild stress model and the olfactory bulbectomized model, LPM570065 demonstrated significant antidepressant-like effects (Zhang et al., 2014; Zhu et al., 2021). In a phase II clinical study, LPM570065 extended-release tablet was safe, well-tolerated, and effective in improving depression symptoms in MDD patients (Mi et al., 2021), suggesting that LPM570065 could be a useful treatment option for MDD patients.

This study was designed to examine the antidepressant-like effects of LPM570065 in a mouse “two-hit” model, a model that is known to induce extensive transcriptional and epigenetic changes in the brain (Peña et al., 2017; Reshetnikov, et al., 2021), and further examine the underlying epigenetic mechanism of antidepressant action.

## Materials and Methods

### Animals

Adult male and female C57BL/6J mice (20–22 g) were purchased from Jinan Pengyue Experimental Animal Center (license number: SCXK20190003). All animals were acclimated to the laboratory environment for at least 5 days before the start of the experiments. Animals were housed in sterile cages under standard conditions (21°C, 50 ± 10% relative humidity, 12/12 h light/dark cycle, food and water ad libitum). The standard protocol was followed for animal mating, and breeding to generate litters. All behavioral experiments were conducted during the animals’ light cycle and in accordance with the National Institutes of Health Guide for the Care and Use of Laboratory Animals (8th edition, 2011) and approved by Yantai University Laboratory Animal Care and Use Committee. Experimenters were blind to experimental groups and drug treatments. Protocol was approved by the Animal Ethics Committee of Yantai University (registration number is YTU20200226) (Yantai, China).

### Drugs

LPM570065 (> 99.93% pure, white powder) was provided by State Key Laboratory of Long-acting and Targeting Drug Delivery Technologies (Yantai, China). The purity of the compound was verified by HPLC. Fluoxetine hydrochloride was purchased from Sigma-Aldrich (St. Louis, MO, United States). LPM570065 was suspended with 0.5% sodium carboxymethylcellulose (SCMC). Fluoxetine hydrochloride was suspended with 0.5% SCMC.

### Experimental Design

**FIGURE 2:**
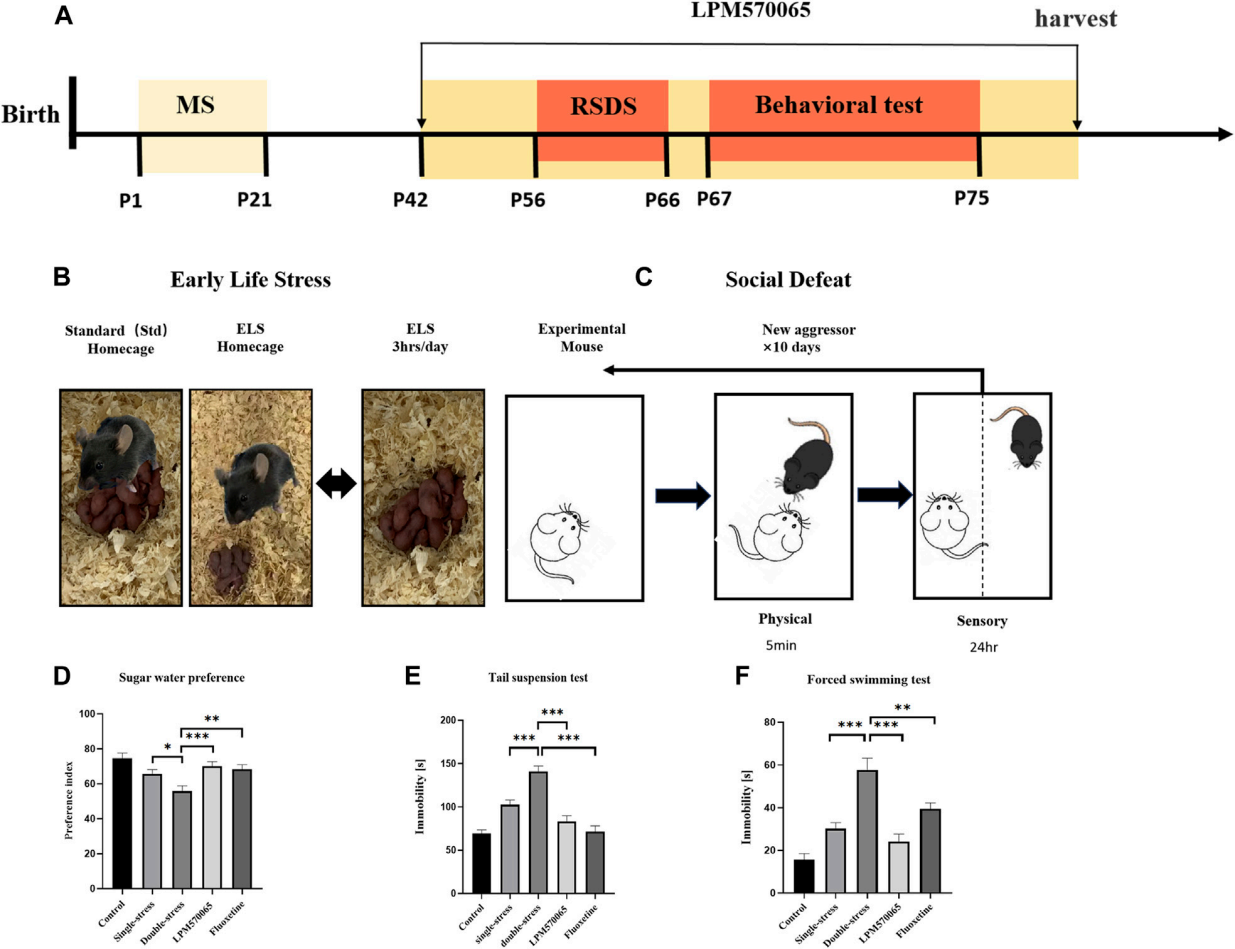
LPM570065 reversed the increased susceptibility to depression in mice experiencing “two-hit” stress. **(A)** A schematic of mother-infant separation. **(B)** A schematic of repeated social defeat stress. **(C)** Experimental timeline. **(D)** Results of sucrose preference test. **(E)** Results of tail suspension test. **(F)** Results of forced swimming test. One-way ANOVA with Bonferroni multiple comparison correction and Student‘s *t* test. Data are mean ± SEM (*n* = 24 mouse per group), and statistical analysis is shown in Supplementary Data. *p < 0.05, **p < 0.01, ***p < 0.001 vs. Double stress group.

A schematic description of the experimental timeline was shown in Figure 2A. Male mice were randomly assigned into 5 groups (*n* = 24 per group): Control group [mice subjected to neither stress and later received vehicle (1 ml/kg) treatment], Single-stress group (mice only subjected to social defeat stress in adulthood and later received vehicle treatment), Double-stress group [mice subjected to double stress (both maternal separation stress and social defeat stress) and later received vehicle treatment], LPM570065 group [mice subjected to double stress and later received LPM570065 (64 mg/kg) treatment], fluoxetine group [mice subjected to double stress and later received fluoxetine (12 mg/kg) treatment]. The vehicle or drug was given by intragastrical administration (i.g.), starting from postnatal day [PND] 42, mice would receive vehicle (0.5% SCMC, i.g.), LPM570065 (64 mg/kg, i.g.) or fluoxetine (12 mg/kg, i.g.) twice daily for the duration until harvest (Figure 2A). The dose of fluoxetine (12 mg/kg) was chosen based on previous studies (Sun et al., 2019), which showed significant behavioral effects.

## Maternal Separation Stress

The maternal separation protocol was as previously described (El Khoury et al., 2006; Musazzi et al., 2010). Briefly, the mouse pups were separated from their dams and moved to separate home cages for 3 h per day during PNDs 1–21 days (Figure 2B). Other than the separation period, the pups were cared for by their dams all the time.

### Repeated Social Defeat Stress

A standard protocol for repeated social defeat stress was followed as described (Golden et al., 2011). Briefly, male CD-1 mice were screened for aggressiveness according to the established criteria (Golden et al., 2011). Adult male C57BL/6J (8 weeks old) mice were subjected to 10 daily, 10 min defeats by a male CD-1 aggressor mouse and were then housed across a plexiglass divider to allow for sensory contact for the remainder of the day. Attack latency, duration, and frequency were not different among groups. Control mice were housed in cages separated from other control mice by a plexiglass divider and were rotated to a different cage daily (Figure 2).

### Behavioral Tests

#### Sucrose Preference Test

The sucrose preference test was performed as previously described with minor modifications (Liu et al., 2018). Briefly, before the test, the individually housed mouse was habituated to consume 1% sucrose solution for 72 h. Animals were deprived of food and water for 12 h and then provided with leakproof bottles containing 1% sucrose or water, randomly placed on the left or right side of the cage. The volume of liquid before and after the 12-h test was weighed to evaluate sucrose and water consumption (*n* = 24). Sucrose preference (%) was calculated as.

Sucrose preference (%) = sucrose consumption (g)/[sucrose consumption (g) + water consumption (g)] × 100%.

#### Tail Suspension Test

The TST was performed in a quiet test room according to published literature with minor modifications (Cryan et al., 2005). Briefly, a four-compartment tail suspension chamber was used, and mice were suspended at 1–2 cm from the tail tip on iron rings with adhesive tape at a height of 35 cm from the table in the middle of the compartment, without contact with the wall. Mice were videotaped for 6 min and the videos were later analyzed by trained experimenters blind to drug treatments wherein the immobility times of the mice during the final 4 min of the 6-min test were recorded and analyzed.

#### Forced Swimming Test

The FST was performed in a quiet test room as described previously with minor modifications (Petit-Demouliere et al., 2005; Dang et al., 2009). Briefly, each mouse was individually placed in a vertical Plexiglas cylinder (40 cm high, 20 cm diameter) containing warm water (25 ± 1°C) at a depth of 20 cm. On the first day of the experiment, the mice were placed in the cylinder for 15 min, and 24 h later they were placed back into the cylinder for a 6 min test. During the test, the mice were videotaped for later scoring by two experimenters blind to the treatment conditions. The immobility times of the mice during the final 4 min of the 6-min test were recorded and analyzed. The inter-rater reliability was 0.93.

### Golgi Staining

Golgi staining was performed according to the manufacturer’s instructions for the FD Rapid Golgi Staining Kit (FD NeuroTechnologies, MD, United States) (Li et al., 2019). Briefly, fresh mouse brains were treated with impregnating solutions (A and B) and stored in total darkness for 2 weeks. The brains were transferred to solution C and stored for 72 h. Brains were cut to 150 µm thickness in a freezing microtome (Thermo Fisher Scientific, United States) and the hippocampal sections were mounted on gelatin-coated microscope slides (Hitobiotec Group, Kingsport, United States). The sections were then immersed in a mixture of solutions D and E. After elution and dehydration, the sections were coated with resin mounting medium. The number of spines of hippocampal CA1 neurons was counted every 10 μm on 40 μm dendritic segments in hippocampal slices using a German ZEISS microscope. Three mice per group were used for brain area sectioning, and three neurons per brain section per mouse were selected for quantification using a double-blind method.

### RNA-Seq and Data Analysis

The high-throughput RNA sequencing analysis for this study was provided by a commercial service (Biotech Biotechnology Inc, Shanghai, China). First, total RNA was extracted from the hippocampal and the VTA tissue of four groups of mice (three samples per group): control group, single-stress group, double-stress group, and LPM570065 group, respectively. The RNA quality and quantity were then analyzed using the Quantum Bit RNA Detection Kit and the Quantum Bit 2.0 Fluorometer (Life Technologies, CA, United States).

### Reduced Representation Bisulfite Sequencing

For the control group, single-stress group, double-stress group, and LPM570065 group (two samples per group), RRBS library creation and heavy sodium sulfite transformation was performed, followed by sequencing analysis. Briefly, total DNA from mouse hippocampal and VTA tissue were extracted separately using the QIAamp Fast DNA Tissue Kit (Qiagen, Düsseldorf, Germany) according to manufacturer’s instructions, followed by sodium sulfite transformation and sequencing using an Illumina Novaseq™ 6000 instrument. Differentially methylated regions (DMRs) were identified using default parameters (sliding window analysis, size 1,000 bp, 500 bp overlap, *p* < 0.05).

### Validation of Methylation Using Pyrophosphate Sequencing Technology

We performed pyrophosphate sequencing based on the results of previous RNA-seq and RRBS sequencing results from the hippocampal tissue to further validate the results, and analyzed the methylation levels of differential genes in the promoter regions of depression susceptibility-related genes between the single- and double-stress groups, and between the double-stress group and the LPM570065 group. The VTA tissue was not analyzed here as previously combined sequencing (RNA-seq and RRBS sequencing) failed to identify genes that met the screening criteria. The kit (B518251, Sangon, Shanghai, China) was used to extract gDNA from the mouse hippocampal samples, followed by the bisulfite transformation step according to the EZ DNA methylation-gold™ kit instructions (D5005, Zymo Research, CA, United States). The DNA was then subjected to PCR and the corresponding genes were sequenced by pyrophosphate using the PyroMark Q48 System (Qiagen) following the instructions of the PyroMark Q48 Advanced CpG reagent (974022, Qiagen). The following genes were analyzed: CLIP associating protein 1 (*Clasp1*), potassium large conductance calcium-activated channel, subfamily M, alpha member 1 (*Kcnma1*), Kruppel-like factor 4 (gut) (*Klf4*), oxytocin receptor (*Oxtr*), adhesion G protein-coupled receptor A2 (*Adgra2*), adhesion G protein-coupled receptor A2 (*Sgms1*), adhesion G protein-coupled receptor A2 (*Kcna1*), and primers specific for zinc finger CCCH type containing 12C (*Zc3h12c*), as shown in Primer Supplementary Table S1.

### Western Blotting

Western blotting experiments were performed as previously described (Pillai-Kastoori et al., 2020; Wang et al., 2020). Briefly, mouse hippocampal tissues (*n* = 8 per group) were collected and homogenized in RIPA buffer containing PMSF (1:100). Lysates were then spun at 12,000 rpm for 20 min at 4°C, and supernatant protein levels were assessed via BCA assay (Beyotime, Shanghai, China). Equal protein quantities (50 μg/sample) were separated *via* SDS-PAGE (GenScript, Nanjing, China) prior to transfer to PVDF membranes (Millipore, MA, United States) blocked with 5% milk or 5% bovine serum albumin, and incubated with the primary antibodies β-actin (AF0003, Beyotime), Dnmt1 (D63A6-5032S, Cell Signaling Technology), Dnmt3a (ab188470, Abcam) and were incubated overnight at 4°C. The membranes were washed three times with TBST and protein bands were detected by ECL after incubation with horseradish 20 peroxidase (HRP)-conjugated secondary antibody (#A0216, Beyotime Institute of Biotechnology). Protein bands were quantified using ImageJ, and normalized using β-actin.

### Real-Time PCR

RNA was extracted from mouse hippocampal tissue in each group (*n* = 6 per group) using TRIzol (Invitrogen, CA, United States) according to the manufacturer’s protocol. the quantity and the quality of eluted RNA samples were verified using a spectrophotometer (Nano Drop 2000, Applied Biosystems, California, United States). The quality of all samples of RNA mass (A260/A280) was 1.8–2.0. cDNA was obtained using SPARK script Ⅱ RT Plus Kit (Spark Jade, Shandong, China). RT-qPCR analysis was then performed using an ABI 7500 RT-PCR instrument. Normalization was performed with β-actin and three replicate operations were performed for each gene, after which the relative quantitative expression of genes was calculated using the 2^-∆∆CT^ method. All primers in this study were purchased from Biotech Biotechnology Inc (Biotech, Shanghai, China).

### The Primers Used Are as Follows

β-actin forward:5′-GTA AAG ACC TCT A TG CCA ACA-3′ and β-actin reverse:5′-GGA CTC A TC GTA CTC CTG CT-3’;

*Oxtr* forward:5′-TGGCGGTCCTGTGTCTCATACTG-3′and OXTR reverse:5′-CGACATCAGCAACAGCAGGTAGG-3’

### Statistical Analysis

Data were expressed as means ± SEM. One-way ANOVA with Bonferroni multiple comparison correction, or by Student’s *t* test were used for data analysis. If ANOVA revealed significant group differences, post hoc-Bonferroni tests were performed to evaluate group differences. Data were analyzed using SPSS for Windows^®^ version 21.0 (IBM, NY, United States) and GraphPad Prism v 9.0 (GraphPad Software, CA, United States). Results showing *p* < 0.05 were considered statistically significant for all analyses.

## Results

LPM570065 reduced susceptibility to depression-like behaviors in adult mice subjected to maternal separation.

To evaluate depression-like behaviors in mice, including anhedonia and behavioral despair, we performed SPT, TST and FST in mice on PD 67∼75. In SPT, the consumption preference for 1% sucrose solution was significantly lower in the double-stress group compared to the single-stress group (*p* < 0.05), and the preference for 1% sucrose solution was significantly higher in mice treated with LPM570065 (64 mg/kg) and fluoxetine (12 mg/kg) (*p* < 0.01, *p* < 0.001, Figure 2D). In TST, the duration of immobility was significantly increased in mice of the double-stress group compared to the single-stress group (*p* < 0.001) and this was significantly lower in mice treated with LPM570065 (64 mg/kg) and fluoxetine (12 mg/kg) (*p* < 0.001, Figure 2E). In FST, the immobility time was significantly increased in the mice from the double-stress group compared with the single-stress group (*p* < 0.001), while the mice treated with LPM570065 (64 mg/kg) and fluoxetine (12 mg/kg) had a significantly shorter immobility time, suggesting the alleviation of behavioral despair (*p* < 0.01, *p* < 0.001, Figure 2F).

LPM570065 protected against the reduced number of dendritic spines in the hippocampal CA1 of mice subjected to stress.

**FIGURE 3:**
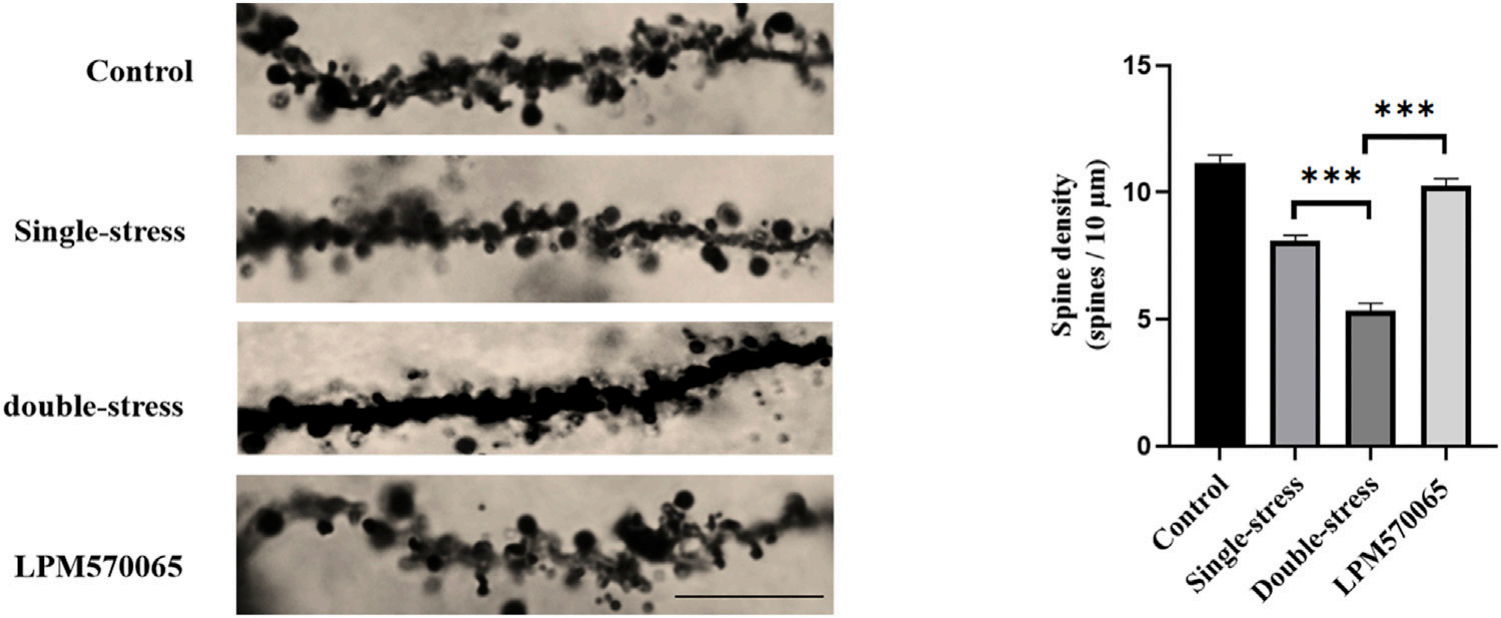
LPM570065 increased the density of dendritic spines in the hippocampus. Dendritic spine density in the hippocampus of mice that experienced early life stress further decreased after a second stressful event in adulthood (*p* < 0.001). The density of dendritic spines in the hippocampus of mice treated with LPM570065 increased (*p* < 0.001). Histograms showed number of dendritic spines per 10 µm of dendrite length for hippocampal pyramidal neuron. One-way ANOVA with Bonferroni multiple comparison correction. Data are mean ± SEM (*n* = 9 neurons). ***p < 0.001 vs. Double stress group.

Dysregulation of synaptic plasticity in the hippocampal CA1 area is associated with major depression (Golden et al., 2013). As expected, the number of dendritic spines in the CA1 area was significantly lower in the single-stress group compared to the vehicle group and the number was even significantly lower in the double-stress group as compared to both the vehicle group and the single-stress group (*p* < 0.001). Interestingly, LPM570065 (64 mg/kg) treatment significantly protected against the stress-induced reduction of dendritic spines, which was not only higher than the double-stress group, it was similar to the vehicle group (*p* < 0.001, Figure 3), suggesting significant protection. These results suggest that maternal separation stress and repeated social defeat stress were significant adversary events that led to dramatic dysregulation of synaptic plasticity in the hippocampal CA1 area and the triple reuptake inhibitor LPM570065 was able to provide significant protection against neuronal insults related to these adversary events.

### RNA-Seq Analysis of Hippocampal and VTA Tissue in Mice

To investigate the potential epigenetic mechanism of LPM570065 to prevent early-life stress-induced depression susceptibility, we performed RNA-seq analysis on hippocampal and VTA tissue from mice.

**FIGURE 4:**
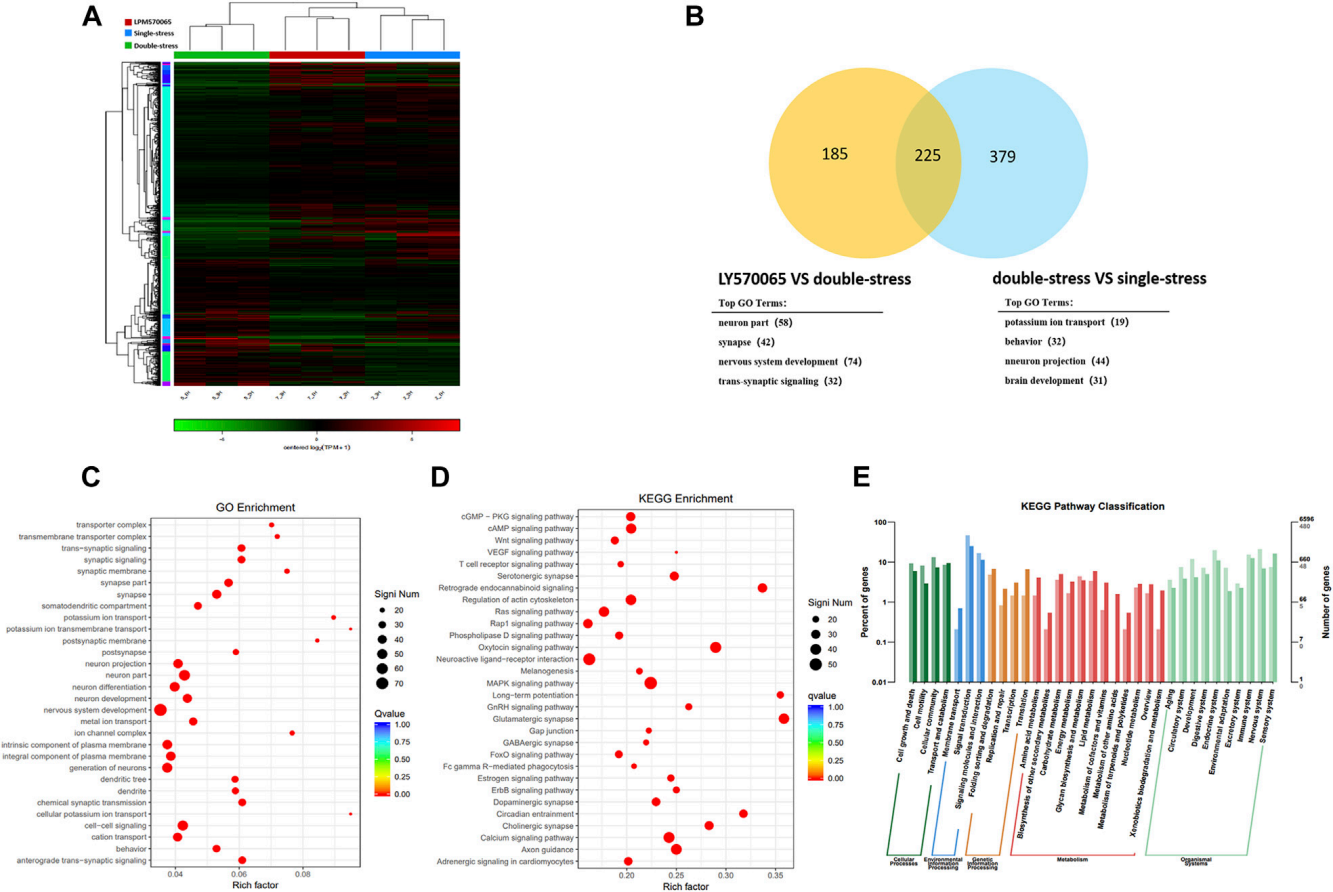
Transcriptome sequencing analysis of the mouse hippocampus and cluster analysis of mouse hippocampal DEGs. For the overall analysis of each group of genes, **(A)** Venn diagram of the clustering analysis of the two compared groups, and gene enrichment GO analysis. **(B)** Clustering heat map of each sample illustrated good inter-group homogeneity and clustering repeatability between samples. **(C)** go function enrichment. **(D,E)** KEGG analysis of differential gene enrichment pathways. (*n* = 3/group, two mice were mixed in each sample, |Fold-change| > 1.5, *p* < 0.05).

The heat map showed clear clustering between samples of different groups (Figure 4A). Transcriptome analysis revealed that repeated social defeat stress altered the expression of 637 genes and double-stress (MS + RSDS) altered the expression of 906 genes as compared to the control group. Cluster analysis showed 604 differentially expressed susceptible genes between double-stress (MS + RSDS) and single-stress (CSDS) groups, and 410 differentially expressed genes (DEGs) between the LPM570065 group and double stress (MS + RSDS) group. The clustering analysis of double stress-single stress *versus* LPM570065-double stress obtained 225 differentially expressed genes (|Fold-change| > 1.5, *p* < 0.05, Figure 4B). All of these 225 DEGs were downregulated in the double-stress (MS + CSDS) group and upregulated in the LPM570065 group (Supplementary Table S2).

Transcriptome analysis of the mouse VTA tissue showed clear clustering between the groups of samples in the heat map (Supplementary Figure S1B). Transcriptome analysis showed that repeated social defeat stress altered the expression of 331 genes and double stress (MS + RSDS) altered the expression of 892 genes as compared to the control group. Cluster analysis yielded 63 differentially expressed susceptibility genes between the double-stress group and the single-stress group, and 778 DEGs between the LPM570065 group and the double-stress group. Cluster analysis of double stress—single stress *versus* LPM570065—double stress identified 29 differentially DEGs. All of these 29 DEGs were downregulated in the double-stress group and upregulated in the LPM570065 group (|Fold-change| > 1.5, *p* < 0.05, Supplementary Figure S1A and Supplementary Table S3).

We next performed enrichment analysis of DEGs in the hippocampal CA1 tissue by the top GO algorithm and functional annotation of the genes revealed that they were associated with synaptic signaling, nervous system development, and other related functions (Figure 4C). KEGG pathway enrichment further revealed that these genes were enriched in endocannabinoid signaling, regulation of oxytocin signaling pathway, and glutamatergic synaptic pathway (Figure 4D), and the annotated classification of the KEGG pathway showed that DEGs were associated with developmental, environmental adaptation, neurological, and other related pathways (Figure 4E).

Enrichment analysis of DEGs in mouse VTA by top GO algorithm and functional annotation of these genes revealed that these genes are associated with developmental and other related functions (Supplementary Figure S1C). KEGG pathway enrichment further revealed that most genes were enriched in Neuroactive ligand-receptor interaction and Rap1 signaling pathway, among other pathways (Supplementary Figure S1D), and the annotated classification of KEGG pathways showed relevance to environmental adaptation, neurological, and other related pathways (Supplementary Figure S1E).

### DNA Methylation Analysis of the Hippocampus and VTA in Mice

**FIGURE 5:**
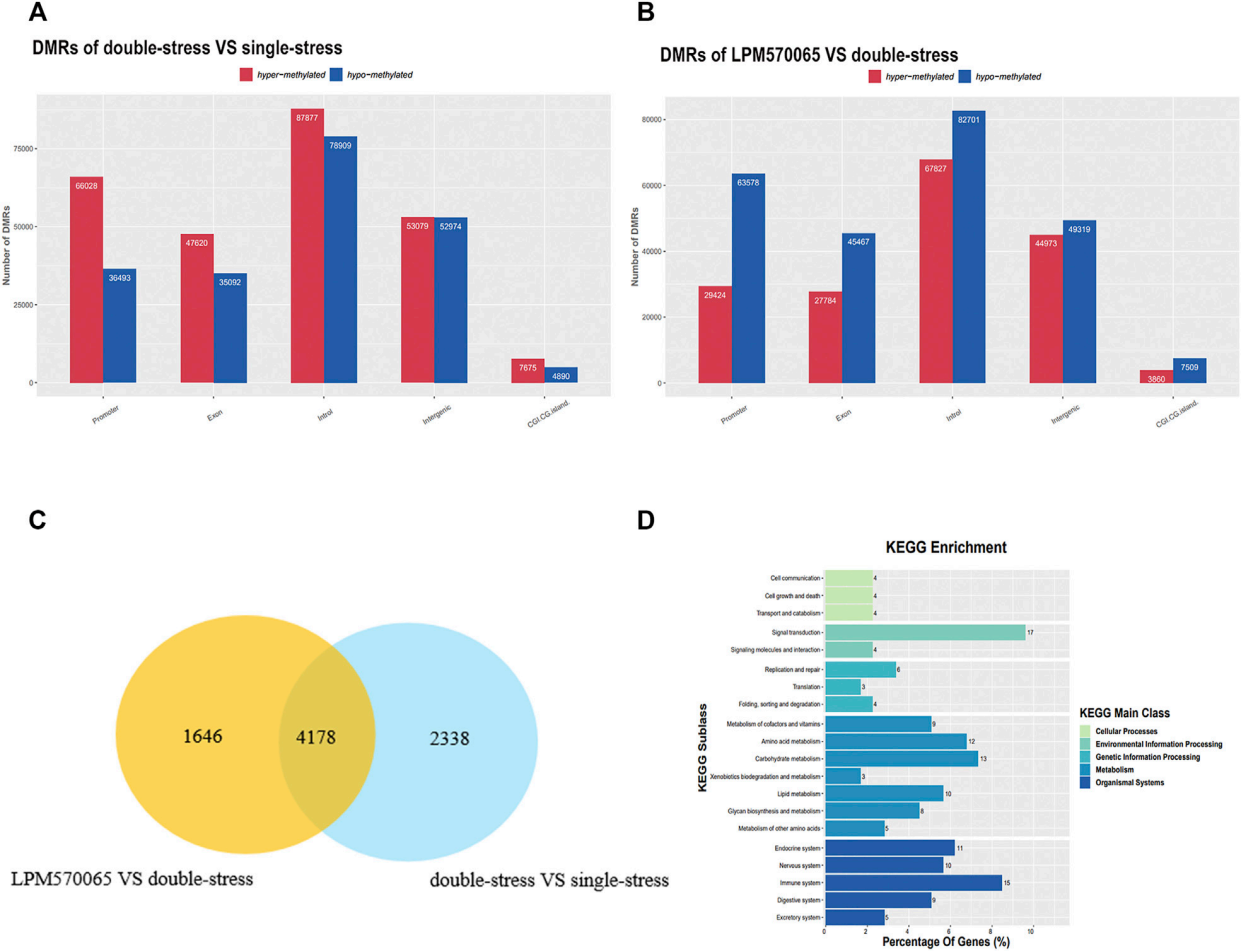
RRBS sequencing for further screening of differential genes for methylation. **(A,B)** Representation in bar graphs of DMRs hypermethylation or hypomethylation in the hippocampus of double stress vs. single stress and LPM570065 vs. double stress mice. (*n* = 2/group, each two mice mixed as one sample, |Fold-change| > 1.5, *p* < 0.05). **(C)** Venn diagram of cluster analysis of the two compared groups identified DMRs between the two compared groups. **(D)** KEGG enrichment analysis, 17% of the genes were found to be enriched in the signaling pathway.

In the DNA methylation analysis, by comparing hippocampal tissue samples from mice in double-stress and single-stress groups, we found that 64% (66,028/102,521) of promoter DMRs exhibited hypermethylation. Comparing the hippocampal tissue samples from mice in the LPM570065 group and double-stress group, we found that 68% (63,578/93,002) of the promoter DMRs exhibited hypomethylation (Figures 5A,B). By cluster analysis of double stress-single stress *versus* LPM570065-double stress, we found that 4,178 promoter DMRs were hypermethylated in the double-stress group and hypomethylated in the LPM570065 group (*p*<0.05, Figure 5C and Supplementary Table S4).

Using the same analysis, by comparing mouse VTA tissue samples from the double-stress group and the single-stress group, we found that 14% (18,486/128,300) of the promoter DMRs exhibited hypermethylation. Comparing mouse VTA tissue samples from the LPM570065 group and the double-stress group, we found that 51% (23,723/46,094) of the promoter DMRs exhibited hypermethylation (Supplementary Figures S2A,B). By cluster analysis of double stress-single stress vs. LPM570065-double stress, we found that 839 promoter DMRs were hypermethylated in the double-stress group and hypomethylated in the LPM570065 group (Supplementary Figure S2C and Supplementary Table S5). KEGG enrichment analysis of genes related to promoter DMRs methylated in double stress-single stress and methylated in LPM570065-double stress was performed in mouse hippocampal CA1 and VTA tissue, respectively (Figure 5D and Supplementary Figure S2D), and both analyses found more genes enriched in signaling (17%) and neurological aspects (10%).

### Changes in Methylation Levels of DEGs in the Hippocampus and VTA

**FIGURE 6:**
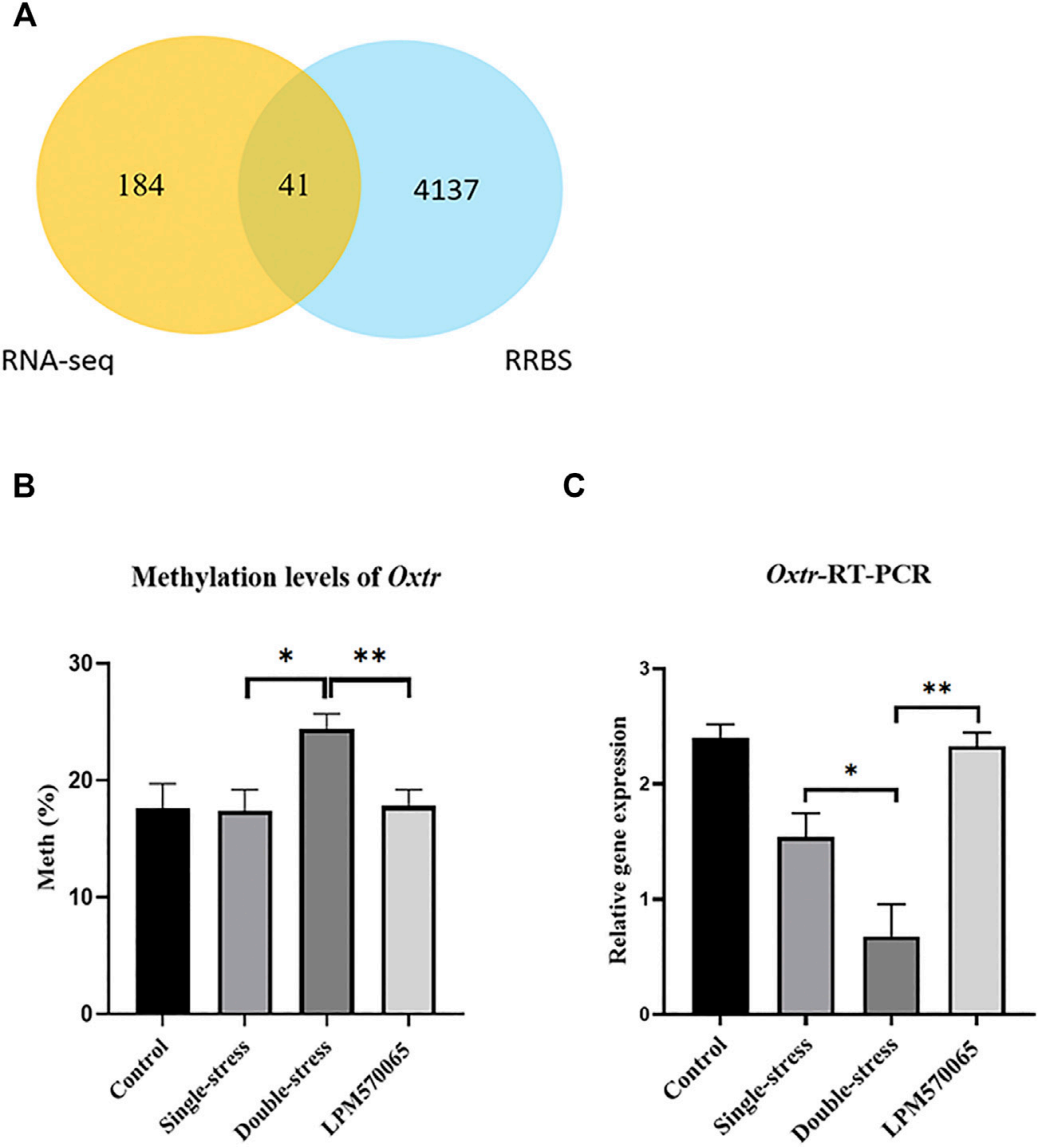
Methylation and validation of DEGs. **(A)** Venn diagram of joint analysis of differential genes screened by RNA-seq and clustered with genes that underwent methylation in RRBS. **(B)** Methylation of gene-specific hippocampal *Oxtr* was assessed by pyrophosphate sequencing in each group of hippocampal tissues. **(C)** Validation of *Oxtr* expression in each group of hippocampal tissues by RT-qPCR. One-way ANOVA with Bonferroni multiple comparison correction. Data expressed as mean ± SEM, *n* = 5–6. *p < 0.05, **p < 0.01, ***p < 0.001 vs. Double stress group.

In the combined analysis of differentially expressed genes and methylation genes in the mouse hippocampal tissue, among the 225 DEGs obtained in the double stress-single stress *versus* LPM570065-double stress clustering analysis, 41 of them had DMRs in the promoter region (|Fold-change| > 1.5, *p* < 0.05, Figure 6A), and their methylation levels were negatively correlated with the gene expression in the double-stress group, i.e., hypermethylation and down-regulation in the double-stress group and hypomethylation and up-regulation in the LPM570065 group. Interestingly, these 41 genes were largely associated with the regulation of potassium channels, G proteins, synapses, synaptic transmission, and oxytocin (Supplementary Table S6). However, with the samples from VTA tissue, no gene met the screening criteria after co-analysis, as such further studies were performed only with hippocampal tissue from these mice.

Validation of gene expression and methylation of depression susceptibility-related genes in the mouse hippocampus.

Among the 41 related genes, there were 8 depression-related genes (*Clasp1, Klf4, Oxtr, Adgra2, Sgms1, Kcna1, Zc3h12c*) (Table 1) with which genetic validation was performed in the double-stress group with low expression of hypermethylation and LPM570065 group with high expression of hypomethylation by RT-PCR and pyrophosphate sequencing. The results showed that the gene *OXTR* was hypo-expressed but hypermethylated in the double-stress group and hyper-expressed but hypomethylated in the LPM570065 group (*p* < 0.05, *p* < 0.01, *p* < 0.001, Figures 6B,C).

**TABLE 1:** Eight genes exhibiting concordance between patterns of promoter hypermethylation or hypomethylation and gene downregulation or upregulation, respectively.

Gene id	GeneName	Sequencing	Double-stress VS single-stress			LPM570065 VS double-stress		
			log2FC	pValue	result	log2FC	pValue	result
ENSMUSG00000064302	Clasp1	RNA-seq	−1.11	0.0002	down	1.10	0.0032	up
		RRBS	2.28	5.09E-11	hyper-methylated	−0.606	0	hypo-methylated
ENSMUSG00000063142	Kcnma1	RNA-seq	−1.13	0.0006	down	0.93	0.0014	up
		RRBS	1.58	0.0002	hyper-methylated	−1.631	1.78E-07	hypo-methylated
ENSMUSG00000003032	Klf4	RNA-seq	−1.20	0.0005	down	0.75	0.0372	up
		RRBS	3.62	4.55E-49	hyper-methylated	−4.616	2.26E-67	hypo-methylated
ENSMUSG00000049112	Oxtr	RNA-seq	-0.92	0.0032	down	0.84	0.0123	up
		RRBS	2.21	1.05E-14	hyper-methylated	−2.087	8.27E-12	hypo-methylated
ENSMUSG00000031486	Adgra2	RNA-seq	−1.00	4.93E-6	down	0.84	0.0003	up
		RRBS	0.807	0	hyper-methylated	−0.921	0.0037	hypo-methylated
ENSMUSG00000040451	Sgms1	RNA-seq	−0.79	0.0281	down	0.76	0.0204	up
		RRBS	3.59	1.14E-71	hyper-methylated	−2.920	2.59E-62	hypo-methylated
ENSMUSG00000047976	Kcna1	RNA-seq	−1.01	0.0026	down	0.81	0.0098	up
		RRBS	1.17	3.37E-10	hyper-methylated	−0.599	0.0000747718836970886	hypo-methylated
ENSMUSG00000035164	Zc3h12c	RNA-seq	−0.80	0.0136	down	0.72	0.0079	up
		RRBS	0.84	0	hyper-methylated	−1.420	3.56E-06	hypo-methylated

LPM570065 regulated the expression of DNA methyltransferases (DNMTs) in the mouse hippocampus.

**FIGURE 7:**
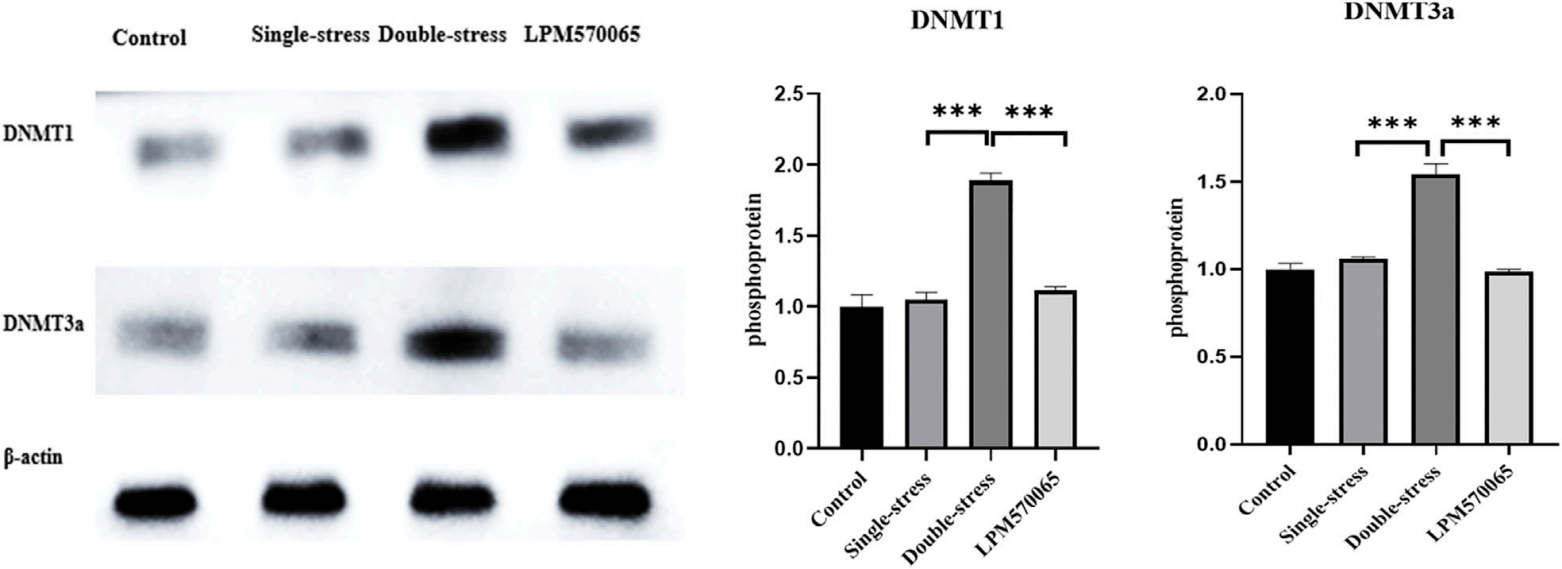
DNMT1 and DNMT3a protein expression in hippocampus. One-way ANOVA with Bonferroni multiple comparison correction. Data are normalized to controls and are expressed as means ± SEM (*n* = 8/group) ***p < 0.001 vs. Double stress group.

Methyltransferases (DNMT) play an important role in the maintenance of methylation in DNA replication and repair. To evaluate the effects of early life stress and LPM570065 on DNMTs, the protein expression levels of DNMT1 and DNMT3a were studied. The expression of both DNMT1 and DNMT3a proteins was significantly higher in the double-stress group compared to the single-stress group (*p* < 0.001) while the expression of both DNMT1 and DNMT3a proteins was significantly reduced after LPM570065 treatment (*p* < 0.001) (Figure 7).

## Discussion

Environmental factors following early traumatic stressful experiences are thought to be important triggers of behavioral abnormalities and psychiatric disorders. Early life stressors (especially ELS) can leave “scars” on the brain, leading to increased susceptibility to depression in later life through epigenetic mechanisms (Torres-Berrío et al., 2019). Although the effects of 5-HT reuptake inhibitors such as fluoxetine, escitalopram and other antidepressants have been studied in relation to their epigenetic mechanisms of antidepressant actions (Chmielewska et al., 2019), little is known of the epigenetic mechanisms of 5-HT/NE/DA triple reuptake inhibitor antidepressants in mediating their antidepressant actions. Results from this study show that when mice that experienced early life stress events (maternal-infant separation in this case) are exposed to social failure (repeated social defeat stress) in adulthood, their depression susceptibility was significantly increased, along with impaired neurogenesis and reduced hippocampal dendritic spine density. Importantly, we found that treatment with the triple reuptake inhibitor LPM570065 significantly improved the depression-related behavioral and morphological changes. Through epigenetic mechanistic analysis, we identified a differentially expressed methylation gene, *Oxtr*, in the mouse hippocampus. *Oxtr* expression was reduced in mice experiencing double stress and this was negatively correlated with the degree of DNA methylation. Importantly, LPM570065 was able to significantly ameliorate depression susceptibility in mice experiencing double stress and this effect was at least partially mediated by reversing the methylation of *Oxtr*. Together, this study extended previous results of the antidepressant-like activity of the triple reuptake inhibitor LPM570065 by revealing a novel epigenetic mechanism of antidepressant actions of this new and potentially important antidepressant drug.

Clinical studies have shown that early life stress increases the incidence of depression later in life (Williams et al., 2016). Preclinical studies have used the combination of early life stress with secondary stress event in adulthood to create a behavioral phenotype that mimics human depression vulnerability (Han et al., 2019). For example, experiencing the “first hit” of maternal separation stress during early life makes an individual more vulnerable to the “second hit” of stressful events in adulthood such as repeated social defeat stress (Peña et al., 2017). In the present study, we adapted this “two-hit” model of depression vulnerability to evaluate the antidepressant-like effects of the triple reuptake inhibitor LPM570065. This model incorporated the early life stress event of maternal separation with the later life stressful event of repeated social defeat and this created a behavioral phenotype that mimics many aspects of human depression symptoms such as anhedonia and social despair (Peña et al., 2017). Our previous studies have shown that LPM570065 did not produce statistically significant changes in voluntary locomotor activity in unstressed mice and rats. (Supplementary Tables S7, S8). However, in TST and FST, LPM570065 administration decreased the immobility time in unstressed mice and rats (Supplementary Tables S9, S10). Indeed, the stressed mice demonstrated significantly reduced sucrose preference and increased immobility in the SPT, FST and TST assays. Under this situation, LPM570065 treatment demonstrated significant protective efficacy such that for all the behavioral measures the mice subjected to double stress were not different from the control mice that never experienced the stress. This suggests that LPM570065 was able to protect against impact of stressful events on behavioral normality and ameliorate depression vulnerability. This finding adds to the previous preclinical and clinical studies supporting the antidepressant efficacy of LPM570065 and suggests that triple reuptake inhibitors in general, and LPM570065 in particular, could be useful new tools to combat against depression.

In an effort to interrogate the potential mechanisms of antidepressant actions of LPM570065beyond its direct monoaminergic reuptake inhibition effects, we examined its effects on neuroplastic and epigenetic changes induced by stressful events. Neuroplasticity is a fundamental mechanism of neuronal adaptation, and chronic stress can induce or exacerbate depression and disrupt neuroplasticity (Pittenger and Duman, 2008). Altered dendritic spines are strongly associated with depression (Ménard et al., 2016; Ammala et al., 2019; Huang et al., 2019). The CA1 region in the hippocampus has been one of the most extensively studied brain regions in depression research (Ma et al., 2021). Patients with MDD show a marked reduction in left CA1 volume (Roddy et al., 2018). Our results show that dendritic spine density in the hippocampal CA1 region is markedly reduced in the double stress model compared to the single stress model, which may be due to the early maternal-infant separation stress prior to the exposure of social defeat stress in adulthood. Most importantly, we found that LPM570065 treatment prevented the dendritic spine density decrease. These results suggest that LPM570065 may be able to prevent deleterious synaptic plasticity maladaptation and subsequently reduce the development and demonstration of depressive-like behaviors.

RNA-seq technology provides crucial information on depression-related pathways and regulatory mechanisms, and RBSS is able to identify DNA methylation patterns associated with specific genes in the brain. Here we adopted a combination of RNA-seq and RRBS to search for DEGs in the mouse hippocampus. This effort led to the identification of *Oxtr*, a gene known to be closely associated with early parental care, depression and their interactions (Cataldo et al., 2018), the promoter methylation status of which was negatively correlated with the observed gene expression pattern. Here we found that *Oxtr* was a DEG in the hippocampus between mice experiencing double stress (maternal separation and repeated social defeat) and those only experienced single stress (repeated social defeat). This is consistent with the established relationship between *Oxtr* expression and early parental care (Cataldo et al., 2018). Importantly, LPM570065 was able to reverse this methylation status, suggesting that the antidepressant efficacy of LPM570065 may be associated with its effect on *Oxtr* gene alterations.

*Oxtr* contains seven transmembrane domains and belongs to the class 1 family of G protein-coupled receptors. The oxytocinergic system plays a key role not only in shaping social behaviors (e.g., trust, social support) but also in regulating responses to stressors (Olff et al., 2013). There is growing evidence that the central 5-HT and oxytocin (OT) systems are closely related and that 5-HT may affect social behavior (e.g., socialization, aggression, depression) through OT release. Studies also show that DA induces OT release (Melis et al., 1989) and that OT-DA interactions are mediated by specific types of DA receptors (D2-DA receptor) in the regulation of social bonding (Liu and Wang, 2003). There is also evidence supporting the interaction between NE and oxytocin, with oxytocin release being regulated by NE in the hypothalamic-neurophysical system (Salmina et al., 2010). Collectively, here we hypothesize that increased *Oxtr* methylation may contribute to the maintenance of early life stress-induced depression susceptibility in adult mice, and that LPM570065, a novel and potent 5-HT/NE/DA triple reuptake inhibitor, may exert its antidepressant effects by reducing *Oxtr* methylation to reverse depression susceptibility.

Methyltransferases (DNMT) play an important role in maintaining methylation in DNA replication and repair (Lyko, 2018). Changes in DNMT expression may be mechanistically related to the changes in DNA methylation in the promoter regions of stress- and depression-related genes (Boersma et al., 2014) such as P11 (Melas et al., 2012). Here we detected a significant increase in the protein expression levels of DNMT1 and DNMT3a in the mice from double stress group, which showed a positive correlation with the methylation levels of the gene *Oxtr*, and, interestingly, LPM570065 treatment reduced the expression of DNMT1 and DNMT3a. These results suggest that LPM570065 might reverse the methylation of *Oxtr* and reduce depression susceptibility in the mice by modulating the expression of DNMT1 and DNMT3a.

In conclusion, our findings suggest that methylation specificity of the gene *Oxtr* may play an important role in early life stress-induced susceptibility to depression in adult mice. LPM570065, a novel 5-HT/NE/DA triple reuptake inhibitor, can reverse the methylation of gene *Oxtr*, thus reducing the susceptibility to depression in mice with experience of early life stress. These results extend previous preclinical and clinical studies of the demonstrated antidepressant efficacy and further support that LPM570065 could be a useful therapy for MDD.

## Data Availability

The data presented in the study are deposited in the NCBI SAR repository, accession number PRJNA801464.
