# Supplementary material for: Epigenetic Mechanism of 5-HT/NE/DA Triple Reuptake Inhibitor on Adult Depression Susceptibility in Early Stress Mice
Source: Front Pharmacol. 2022 Mar 17;13:848251. doi: 10.3389/fphar.2022.848251 (PMC8968447; doi:10.3389/fphar.2022.848251)
Supplement: Supplementary file 1 [file DataSheet1.zip › Table 1.DOCX]

|  |  |  | Double-stress VS Single-stress | | | LPM570065 VS Double-stress | | |
| --- | --- | --- | --- | --- | --- | --- | --- | --- |
| Gene id | GeneName | Sequencing | log2FC | pValue | result | log2FC | pValue | result |
| ENSMUSG00000064302 | Clasp1 | RNA-seq | -1.11 | 0.0002 | down | 1.10 | 0.0032 | up |
|  |  | RRBS | 2.28 | 5.09E-11 | hyper-methylated | -0.606 | 0 | hypo-methylated |
| ENSMUSG00000063142 | Kcnma1 | RNA-seq | -1.13 | 0.0006 | down | 0.93 | 0.0014 | up |
|  |  | RRBS | 1.58 | 0.0002 | hyper-methylated | -1.631 | 1.78E-07 | hypo-methylated |
| ENSMUSG00000003032 | Klf4 | RNA-seq | -1.20 | 0.0005 | down | 0.75 | 0.0372 | up |
|  |  | RRBS | 3.62 | 4.55E-49 | hyper-methylated | -4.616 | 2.26E-67 | hypo-methylated |
| ENSMUSG00000049112 | Oxtr | RNA-seq | -0.92 | 0.0032 | down | 0.84 | 0.0123 | up |
|  |  | RRBS | 2.21 | 1.05E-14 | hyper-methylated | -2.087 | 8.27E-12 | hypo-methylated |
| ENSMUSG00000031486 | Adgra2 | RNA-seq | -1.00 | 4.93E-6 | down | 0.84 | 0.0003 | up |
|  |  | RRBS | 0.807 | 0 | hyper-methylated | -0.921 | 0.0037 | hypo-methylated |
| ENSMUSG00000040451 | Sgms1 | RNA-seq | -0.79 | 0.0281 | down | 0.76 | 0.0204 | up |
|  |  | RRBS | 3.59 | 1.14E-71 | hyper-methylated | -2.920 | 2.59E-62 | hypo-methylated |
| ENSMUSG00000047976 | Kcna1 | RNA-seq | -1.01 | 0.0026 | down | 0.81 | 0.0098 | up |
|  |  | RRBS | 1.17 | 3.37E-10 | hyper-methylated | -0.599 | 0.0000747718836970886 | hypo-methylated |
| ENSMUSG00000035164 | Zc3h12c | RNA-seq | -0.80 | 0.0136 | down | 0.72 | 0.0079 | up |
|  |  | RRBS | 0.84 | 0 | hyper-methylated | -1.420 | 3.56E-06 | hypo-methylated |
