# Supplementary material for: Epigenetic Mechanism of 5-HT/NE/DA Triple Reuptake Inhibitor on Adult Depression Susceptibility in Early Stress Mice
Source: Front Pharmacol. 2022 Mar 17;13:848251. doi: 10.3389/fphar.2022.848251 (PMC8968447; doi:10.3389/fphar.2022.848251)
Supplement: Supplementary file 1 [file DataSheet1.zip › Table 8.DOCX]

**Table 1. The experimental grouping of mice**

| Group number | Group | Dosage  (mg/kg) | Concentration  (mg/ml) | Number of animals  (n) | Animal number | |
| --- | --- | --- | --- | --- | --- | --- |
|  |  |  |  |  | Female | Male |
| 1 | vehicle | 0 | 0 | 10 | 5 | 5 |
| 2 | Low | 8 | 0.8 | 10 | 5 | 5 |
| 3 | Medium | 40 | 4.0 | 10 | 5 | 5 |
| 4 | High | 200 | 20 | 10 | 5 | 5 |

**Table 2.** **Effect of single gavage administration of LPM570065 on the** **voluntary locomotor activity in mice within10 min** **(±SD)**

| Group | Dosage  (mg/kg) | 30 min | 1 h | 24 h |
| --- | --- | --- | --- | --- |
| vehicle | 0 | 312.9±111.0 | 284.3±71.4 | 242.3±84.2 |
| Low | 8 | 294.8±86.7 | 230.9±85.8 | 254.4±90.2 |
| Medium | 40 | 304.6±85.0 | 203.1±74.7 | 234.4±95.7 |
| High | 200 | 329.9±154.7 | 222.4±55.8 | 273.0±81.5 |

**Table 3. The experimental grouping of rats**

| Group number | Group | Dosage  (mg/kg) | Concentration  (mg/ml) | Number of animals  (n) | Animal number | |
| --- | --- | --- | --- | --- | --- | --- |
|  |  |  |  |  | Female | Male |
| 1 | vehicle | 0 | 0 | 10 | 5 | 5 |
| 2 | Low | 4 | 0.8 | 10 | 5 | 5 |
| 3 | Medium | 20 | 4.0 | 10 | 5 | 5 |
| 4 | High | 100 | 20 | 10 | 5 | 5 |

**Table 4. Effect of single gavage administration of LPM570065 on the number of hind limbs upright in rats (±SD)**

| Group | Dosage  (mg/kg) | Number of animals  (n) | Number of uprightness within 3min | | |
| --- | --- | --- | --- | --- | --- |
|  |  |  | 0.5h | 1h | 24h |
| vehicle | 0 | 10 | 7.6±3.2 | 3.9±4.1 | 2.0±3.1 |
| Low | 4 | 10 | 7.5±5.8 | 5.0±5.5 | 0.6±1.1 |
| Medium | 20 | 10 | 6.7±3.8 | 2.8±3.5 | 2.8±4.3 |
| High | 100 | 10 | 6.8±2.9 | 2.2±2.9 | 2.0±2.4 |

**Table 5. Effect of single gavage administration of LPM570065 on the number of fecal pellets in rats (±SD)**

| Group | Dosage  (mg/kg) | Number of animals  (n) | Number of fecal pellets within 3min | | |
| --- | --- | --- | --- | --- | --- |
|  |  |  | 0.5h | 1h | 24h |
| vehicle | 0 | 10 | 0.1±0.3 | 0.3±0.7 | 1.2±1.4 |
| Low | 4 | 10 | 0.3±0.5 | 0.7±0.9 | 0.4±0.7 |
| Medium | 20 | 10 | 0.0±0.0 | 0.3±0.5 | 0.7±0.8 |
| High | 100 | 10 | 0.1±0.3 | 0.3±0.5 | 1.1±1.3 |


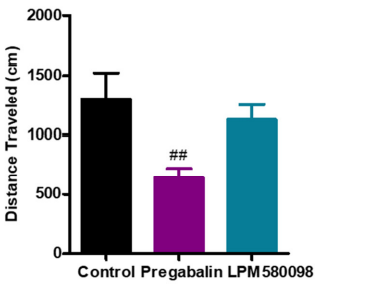


**Figure 1. Effects of LPM580098 on locomotor activity in normal rats.**
