# Supplementary figures and images for: Epigenetic Mechanism of 5-HT/NE/DA Triple Reuptake Inhibitor on Adult Depression Susceptibility in Early Stress Mice
Source: Front Pharmacol. 2022 Mar 17;13:848251. doi: 10.3389/fphar.2022.848251 (PMC8968447; doi:10.3389/fphar.2022.848251)

---

|       |             |
|-------|-------------|
| Group | <i>OXTR</i> |
|-------|-------------|

---

Control

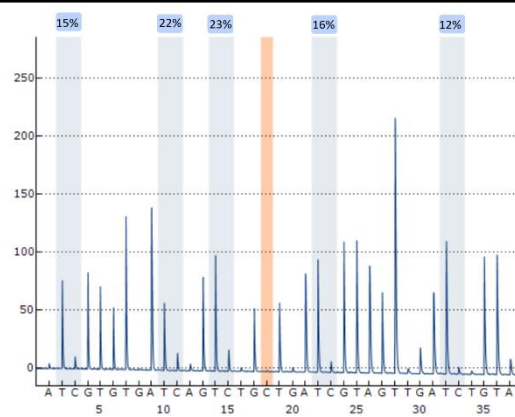

Single-stress

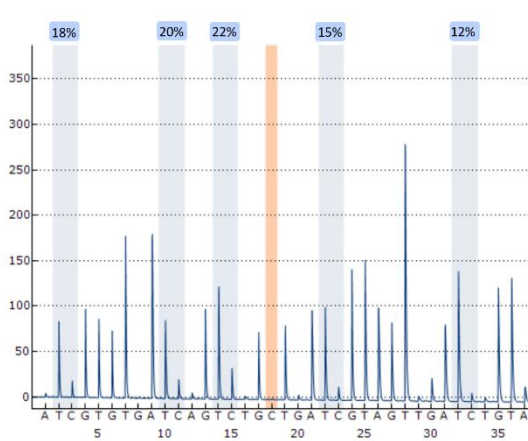

Double-stress

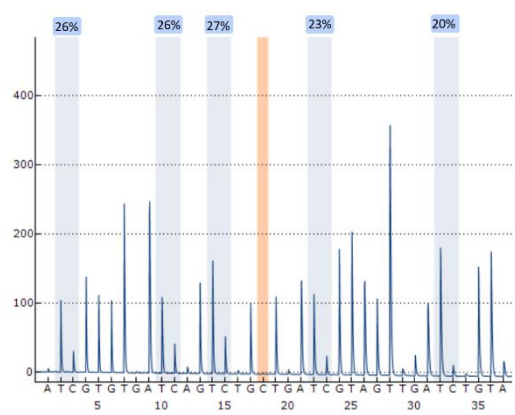

LPM570065(64mg/kg)

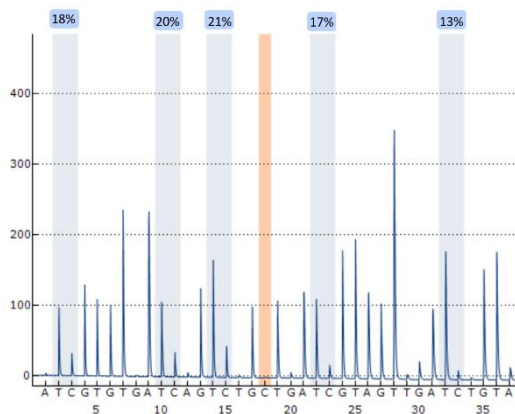

Supplement: Supplementary file 1 [file DataSheet1.zip › Data Sheet 1.PDF]

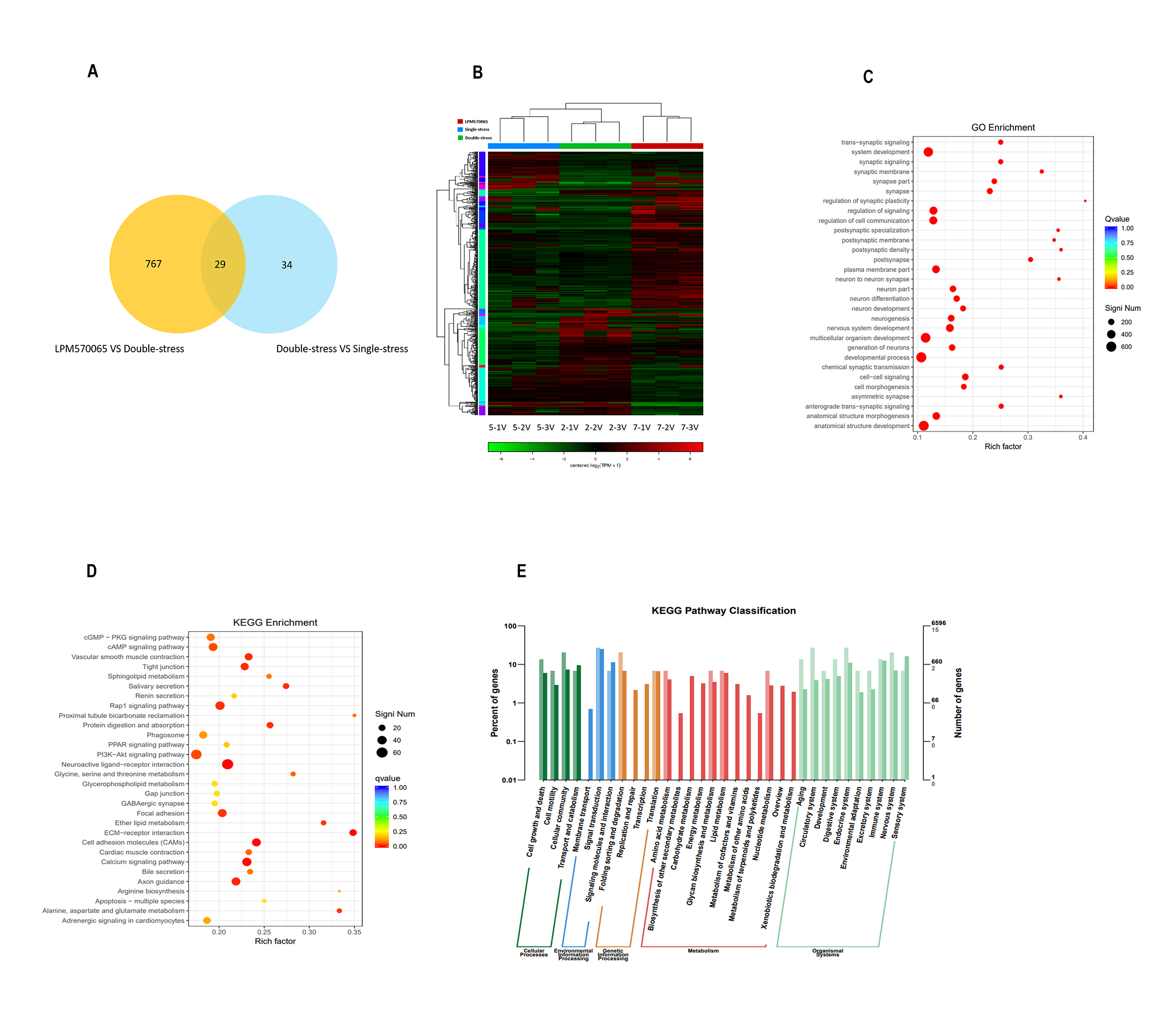

Supplement: Supplementary file 1 [file DataSheet1.zip › Image 1.TIF]

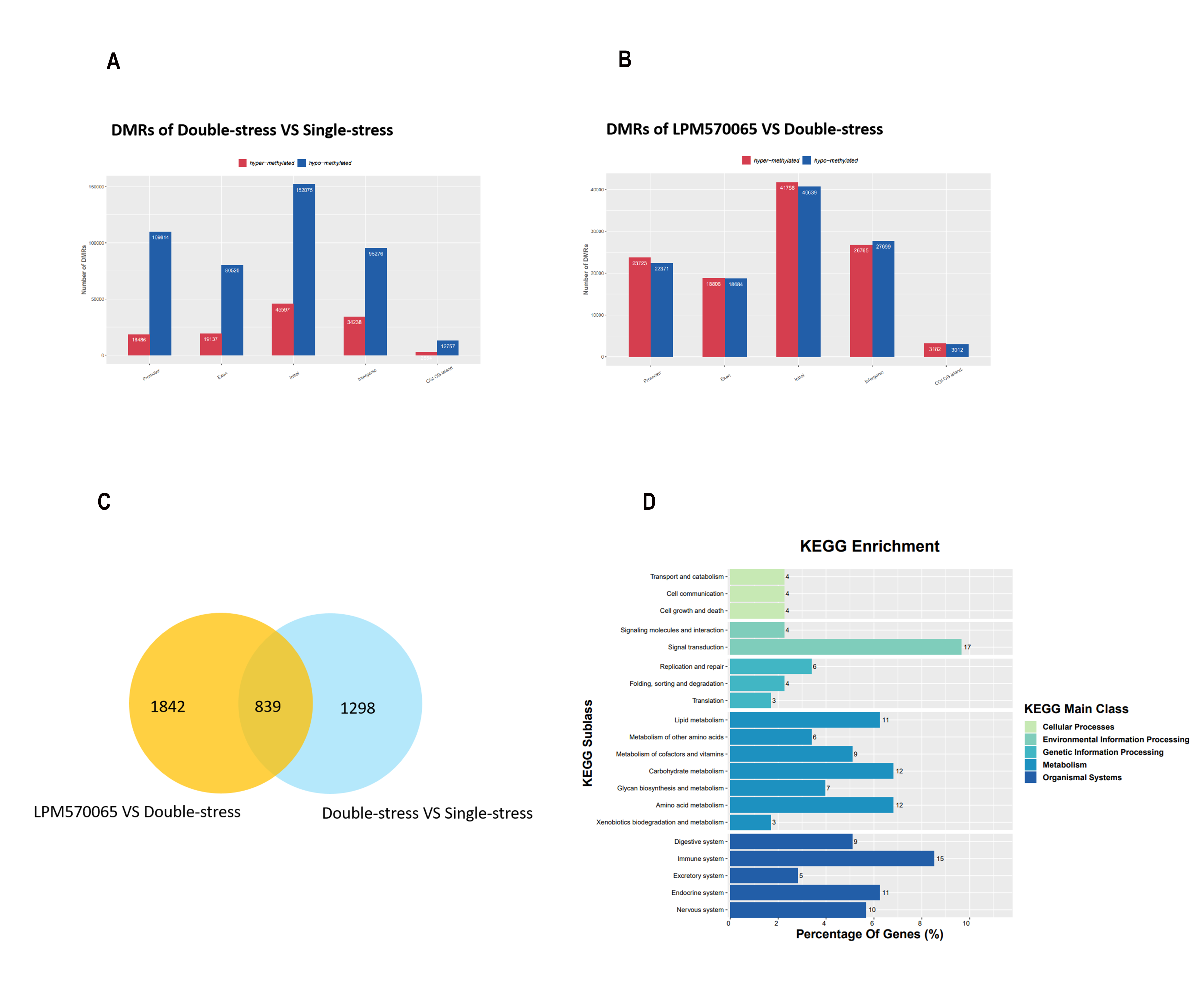

Supplement: Supplementary file 1 [file DataSheet1.zip › Image 2.TIF]

| DNMT1 | DNMT3a | ACTIN |
| --- | --- | --- |
| 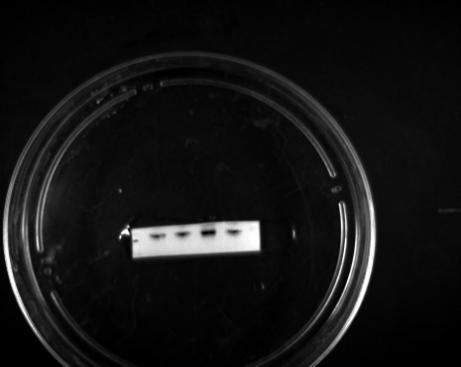 | 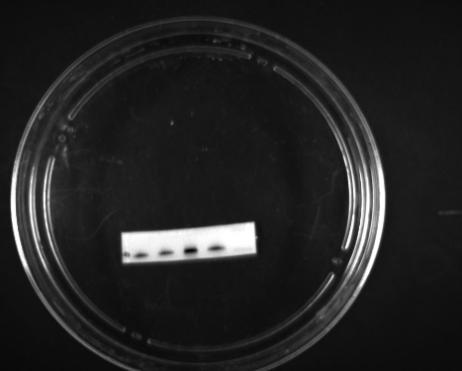 | 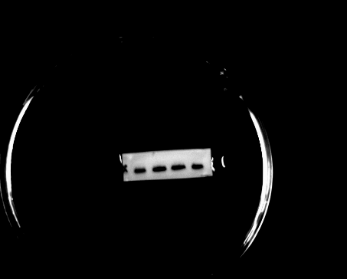 |
| 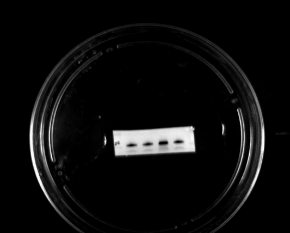 | 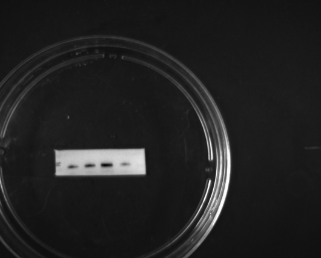 | 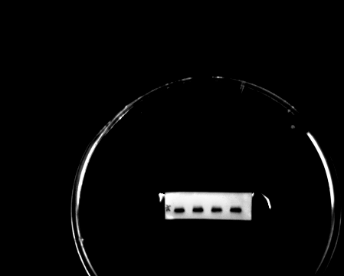 |
| 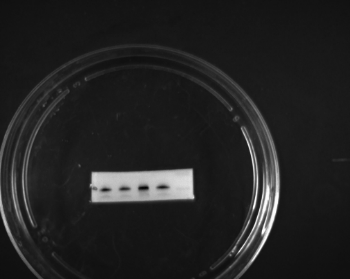 | 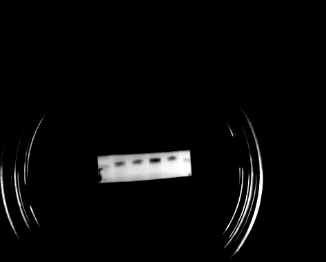 | 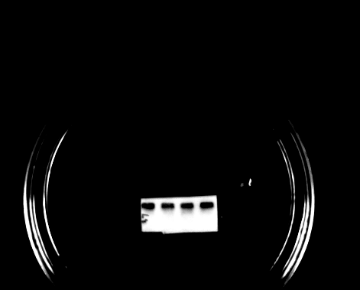 |
| 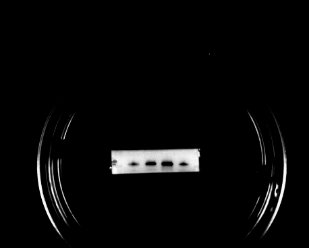 | 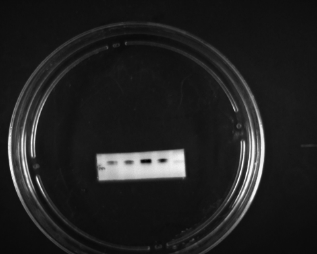 | 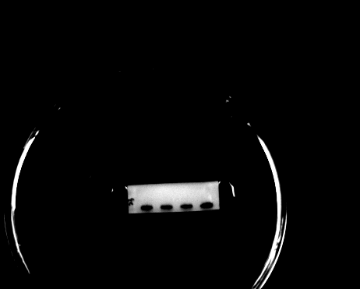 |
| 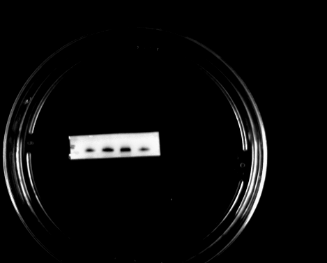 | 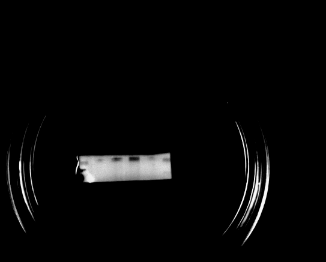 | 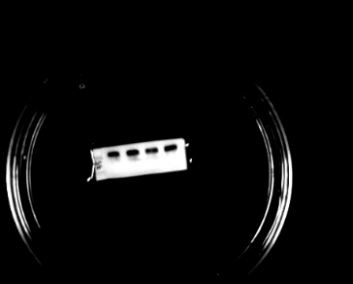 |
| 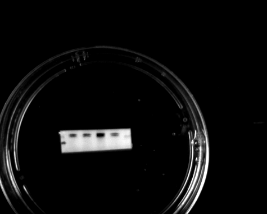 | 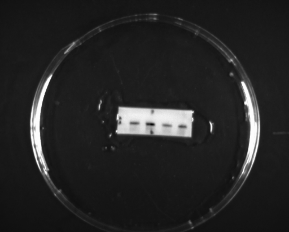 | 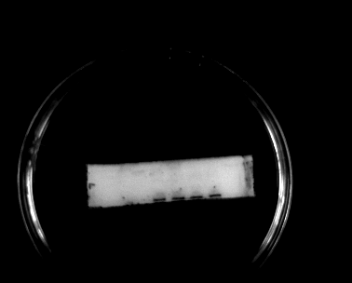 |
| 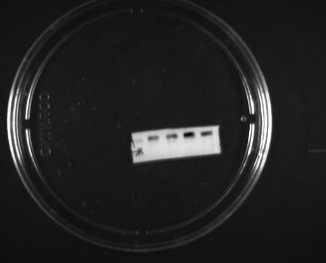 | 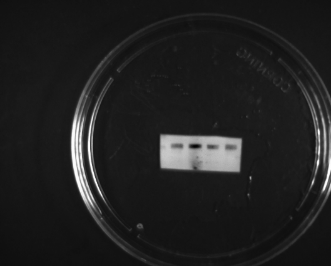 | 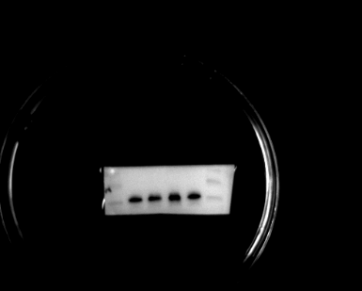 |
| 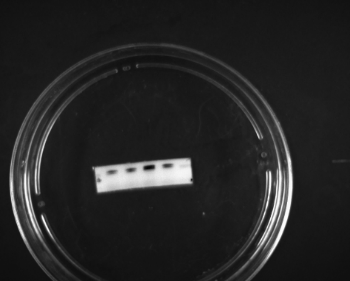 | 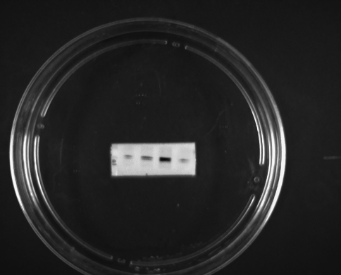 | 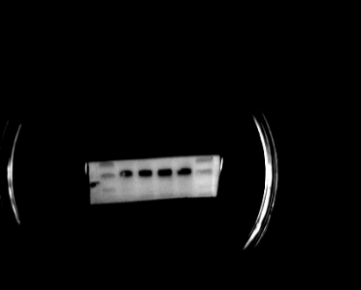 |

Supplement: Supplementary file 1 [file DataSheet1.zip › Table 9.DOCX]
